# Lasers in the management of alopecia: a review of established therapies and advances in treatment

**DOI:** 10.1007/s10103-024-04054-7

**Published:** 2024-04-17

**Authors:** Philippe Jean-Pierre, Anika Pulumati, Eli Kasheri, Melanie Hirsch, Keyvan Nouri

**Affiliations:** 1 Dr. Phillip Frost Department of Dermatology and Cutaneous Surgery, Miller School of Medicine, University of Miami, 1600 NW 10th Ave, 33136 Miami, FL USA; 2 University of Missouri-Kansas City School of Medicine, Kansas City, MO USA

**Keywords:** Androgenetic alopecia, Alopecia Areata, Laser, Hair regrowth

## Abstract

Alopecia, also known as hair loss, is a highly prevalent condition affecting millions of men and women in the United States and worldwide, making it one of the most common complaints by patients presenting to a dermatologist. The symptomology on the presentation of alopecia can be highly variable, ranging from diffuse thinning of hair, discrete and localized patches completely absent of hair, or noticing significant shedding when brushing and showering. Although alopecia does not have a direct negative health impact on patients, it is nonetheless a debilitating disease as it can profoundly impact an individual’s self-image and psychosocial well-being. There are multiple treatment options available to patients with alopecia, and they are typically tailored to the patient’s needs and preferences. The most common of these is the Food and Drug Administration-approved drugs for alopecia, minoxidil, and finasteride. However, both of these are known to be partially efficacious for all patients, so clinicians often use different modalities in conjunction with them, in particular laser-based therapies. This review article will provide a comprehensive assessment of lasers and other light therapies that may be used to manage the two most common types of alopecia: androgenetic alopecia and alopecia areata.

## Introduction

Androgenetic alopecia (AGA), commonly known as male-pattern or female-pattern baldness, is the most prevalent type of alopecia both in the United States and worldwide. Roughly 30–50% of men will have experienced AGA to some degree by the age of 50. AGA is progressive and typically follows a predictable loss of hair in the pattern described on the Norwood scale, beginning with gradual recession at the temples of the scalp, followed by hair loss at the crown of the head [1]. The pathogenesis of the condition is thought to be caused by androgens, particularly dihydrotestosterone (DHT), binding to receptors in the hair follicle and inducing miniaturization [2, 3]. However, studies have shown that susceptibility to AGA is not directly related to serum levels of androgens like testosterone and DHT but rather a genetic predisposition that causes androgen receptors in the hair follicle to be more sensitive to androgens than in other less susceptible patients [4, 5]. Currently, two medications approved by the Food and Drug Administration (FDA) for treating AGA are minoxidil and finasteride [6]. Although these drugs have demonstrated effectiveness in stabilizing and preventing AGA, they require continuous usage and are less effective in reversing the condition. Both carry a risk for notable side effects: finasteride can cause erectile dysfunction and reduced libido in men, as well as potential congenital disabilities in male fetuses from DHT suppression, and this is contraindicated in women of childbearing age [7, 8]. A more definitive treatment for AGA is hair transplantation, a relatively safe procedure with results typically lasting over 20 years. However, the procedure can be cost-prohibitive for many patients. Furthermore, patients usually experience decreased hair density as loss of non-transplanted hair will continue to progress over time [9, 10].

Alopecia Areata (AA) is the second most common cause of alopecia, affecting about 2% of the global population [11]. Unlike AGA, the pathogenesis of AA is unclear, although it has been established to be caused by an autoimmune process [12]. Hair loss in AA typically presents with focal areas of loss, primarily on the scalp, eyebrows, or eyelashes, but may affect any area of the body [12]. The course of AA is also far less predictable compared to AGA, often presenting in intermittent episodes that may wax and wane in severity. Management for AA involves limiting the inflammatory process, so topical and intralesional corticosteroids (ILCS), such as triamcinolone, have classically been first-line [13, 14]. Since 2022, the FDA has approved two Janus Kinase (JAK) inhibitors for treating AA, baricitinib and ritlecitinib. However, both drugs are recent approvals and will require further analysis to assess for long-term effectiveness and side effects [15].

Considering that there are not many highly effective treatments with minimal side effects for the treatment of alopecia, there is a great demand for other novel treatment modalities, often used in conjunction with conventional treatments, to maximize hair regrowth potential. Of these, laser and light therapies have been garnering some of the most interest, as there is a growing body of evidence that they can be highly effective therapeutics in managing alopecia while also often having less significant adverse effects compared to some conventional treatments.

## Lasers in alopecia management

### Low-level light therapy (LLLT)

LLLT is at the forefront of emerging laser-based therapies in treating AGA. LLLT encompasses a range of wavelengths from red to infrared laser light (600–950 nm) and promotes tissue repair and regeneration in a process known as photobiomodulation [16]. The potential for LLLT to stimulate hair regrowth was first observed in 1967, as Mester et al. used LLLT in an attempt to treat cancer in rats with shaved backs, yet unexpected hair regrowth was seen on the backs of the rats [17]. Further studies have since been done, and the phenomenon of hair regrowth under LLLT has been widely acknowledged [18]. Multiple hypotheses have been proposed to explain the precise mechanism of LLLT on hair regrowth, but there has yet to be a clear consensus. LLLT appears to increase hair regrowth by stimulating the anagen reentry of telogen hair follicles, ultimately prolonging the anagen phase of the hair cycle and stimulating the growth of dormant hair follicles [19]. Another proposed explanation behind LLLT is that it can boost hair regrowth by enhancing microvascular circulation, stimulating hair follicles, and increasing tensile strength through anti-inflammatory and antioxidant processes [20].

Leavitt et al. performed the first randomized controlled trial (RCT) to examine the effects of LLLT on hair regrowth in patients with AGA in 2009. 110 participants with AGA were recruited and assigned to receive LLLT or a sham device for 15-minute sessions that were done 3 times a week for a total of 26 weeks. The study found that patients in the LLLT group had significantly more significant mean terminal hair density and total hair regrowth increases. Furthermore, the patients in the LLLT group tolerated the therapy well, and no serious adverse events were reported. This study was sponsored by Lexington International, the company that created the HairMax Lasercomb®, and the successful findings of this study led to the FDA approval of the product [21].

To further support the effectiveness of the newly approved HairMax LaserComb®, in 2014, Jimenez et al. performed a multi-center RCT assessing the effects of LLLT on hair regrowth in AGA patients. 141 female and 128 male patients with AGA were recruited to participate in the study, in which the HairMax Lasercomb® was used. Patients were randomly assigned to the control group involving a sham device or the therapeutic group, which utilized LLLT. The study found that after 26 weeks of receiving three times a week LLLT therapy, the patients in the LLLT group had a significantly higher increase in mean terminal hairs compared to the sham device group. The increase in terminal hair density was found to be independent of the age and sex of the subjects. Furthermore, a higher percentage of subjects treated with the device reported improved hair thickness and fullness on self-assessment than sham-treated subjects [22].

Kim et al. performed a similar study, recruiting 40 subjects in a 24-week RCT. The subjects used an LLLT device emitting 630, 650, and 660 nm wavelengths for 18 min daily and were compared to a sham device group. The LLLT group had significantly greater hair density than the sham device group, and no severe adverse reactions were reported in either group [23].

In 2017, a RCT was done by Esmat et al. to assess the efficacy of LLLT compared to topical 5% minoxidil and a combination of both therapies in managing AGA. The study recruited 45 female patients with proven female pattern hair loss who were followed for 4 months. They were divided into three groups: the first group received only topical 5% minoxidil twice daily, the second group received LLLT for 3 times a week session lasting 25 min, and the third group of patients received a combination of topical 5% minoxidil in addition to the LLLT regimen. The study results were evaluated using ultrasound biomicroscopy (UBM) of the total hair count, clinical assessment, and patient satisfaction. On UBM, the LLLT and combination group demonstrated statistically significant increases in hair count after the 4 months. Using the Ludwig classification, only the combination therapy improved significantly. However, on physician assessment alone, 80–100% of the patients showed clear improvement in all 3 groups. For patient satisfaction, the group that received combination therapy had statistically significantly higher patient satisfaction than the other 2 monotherapy groups. This study is noteworthy as it compares LLLT with a well-known treatment for AGA in minoxidil, and the results show that LLLT is an effective and safe treatment option for AGA with results comparable to minoxidil 5%. Furthermore, the significantly better results yielded in the combination group indicate that combining LLLT with conventional hair loss treatments may be the most effective modality in managing AGA [24].

Across all these studies, most patients with alopecia undergoing LLLT experienced significant hair regrowth while avoiding any major adverse effects, with only a few reporting minor side effects. The most common were headaches, mild pruritus, erythema, and dryness at the application site. These successful findings indicate that LLLT is likely an effective treatment option for AGA, with the option of being combined with other therapies like minoxidil for increased efficacy.

### 308 nm excimer laser

The 308 nm excimer laser is a therapy that has been postulated to be effective for the management of AA, owing to its anti-inflammatory properties that work via apoptosis of T-cells. This laser treats autoimmune dermatologic conditions such as psoriasis and vitiligo, thanks to its immunosuppressive effects [25, 26]. Given the autoinflammatory nature of AA, the excimer laser is the most used laser for treating AA—the laser works by emitting a potent dose of long-wave monochromatic UVB radiation [27, 28].

In 2007, Zakaria et al. were the first to report successful utilization of the excimer laser in the treatment of alopecia, performed in a prospective study involving nine patients. The excimer laser was utilized on patches of hair loss, and one patch on the other side of the scalp was untreated, serving as the control group. Of the patients recruited to the study, all those with AA experienced hair regrowth. The only adverse events reported were hyperpigmentation and erythema in areas directly targeted by the laser. Follow-up 3 months later demonstrated maintenance of hair regrowth [29].

Di Filippo et al. performed a retrospective study with long-term follow-up on patients with AA using 308-nm excimer therapy. 36 patients met the inclusion criteria, and the treatment protocol included a twice-weekly regimen of excimer laser therapy for a total of 12 weeks, targeting patches of hair loss on the scalp. Overall, 52% of the patients experienced significant hair loss regrowth in treated areas. Notably, the patients with ophiasic AA only had 25% hair regrowth. This suggests that specific subtypes of AA are more likely to experience regrowth, whereas others are less likely [30].

Kianfar et al. performed a RCT in 2021 to compare the efficacy and safety of the 308-nm excimer laser with ILCS in the management of AA. 16 patients with at least two patches of AA were randomly assigned to receive either monthly injections of ILCS or weekly treatments of the excimer laser, and the treatment duration was 3 months. Photography and trichoscopy images of the patches were examined at baseline, after the last treatment, and one month after the last treatment. When evaluated at the last treatment session, hair regrowth in the laser group was significantly lower than in the ILCS group; however, when reevaluated 1 month later, the difference in hair regrowth was not statistically significant. The study defined a positive response as over 50% of hair regrowth in the patch and found that 47% of patients who received the excimer laser achieved a positive response, and 66% of patients who received ILCS achieved a positive response. One clear advantage of the excimer laser compared to ILCS in this study was that there were no reported severe adverse events for the excimer laser group.

In contrast, 50% of patients in the ILCS group experienced more severe adverse events, including skin atrophy and hypopigmentation. This study demonstrated that the 308-nm excimer laser was a safe and effective method for hair regrowth in the management of AA. However, hair regrowth may be delayed compared to treatment with ILCS [31].

### 1550-nm nonablative fractional erbium glass laser (Er: glass laser)

Recent studies have suggested that the Er: glass laser effectively stimulates hair regrowth in both AGA and AA patients. Fractional photothermolysis stimulates the Wingless-related integration site 5a (Wnt 5a)/β-catenin signaling pathway, which plays a crucial role in hair regrowth as molecular studies show that when this signal is upregulated, there is an associated increase in the change from the telogen phase to the anagen phase of the hair cycle [32]. Fractional photothermolysis also induces micro-coagulative trauma on the papillary dermis, stimulating wound healing factors that promote hair regrowth, including vascular endothelial growth factor (VEGF) and fibroblast growth factor 7 (FGF7) [33]. The thermal effect from the photomechanical waves stimulates the expansion of the extracellular space in the stratum corneum, allowing for improved drug delivery of topical solutions like minoxidil [34].

Muhsin et al. performed a RCT that enrolled 30 AA patients with two patches of alopecia each for 60 patches. Each patient had one patch enrolled into a control group, which was treated by topical minoxidil alone, and the other patch was assigned to the study group, which was treated with both Er: glass laser and topical minoxidil. 60% of the patients in the study group experienced hair regrowth, but only 16% for the control group. These findings suggest that the erbium glass laser effectively stimulates hair regrowth in AA patients [35]. A limitation of this trial is that all participants received minoxidil therapy. Thus, it cannot be concluded if the laser alone stimulates hair regrowth or if it instead enhances the effects of the minoxidil.

Eckert et al. performed a case series in which the Er: glass laser was used to treat AA in 5 patients. The treatment involved patients receiving the laser therapy in 2–3 sessions at 3–6 weeks intervals. At the 3-month follow-up, all the patients experienced complete or near-complete hair regrowth. These 5 patients were monitored for the subsequent 2–4 years, and none of them experienced recurrence of the original patches of hair loss, although one patient did report new patches of hair loss. The laser therapy was well-tolerated, and the only adverse effect throughout the treatment was some pain at the site targeted during the laser therapy [36].

Kim et al. performed a pilot study to evaluate the effect of the Er: glass laser on AGA in 20 male participants. The participants each received a laser treatment every 2 weeks over a 10-week period. No significant increase in hair regrowth was observed; however, both the patients and clinicians endorsed clinical improvements in the hair density and growth rate for all the subjects [37].

The current literature indicates that the Er: glass laser may be an effective therapy for promoting an increase in hair density and growth via an increase of the anagen: telogen phase ratio. Combining the laser treatment with already established treatments has a synergistic effect on hair regrowth, suggesting that the fractional erbium-glass laser can work well as an adjunctive therapy for AGA and AA patients already using medications like minoxidil. Further studies should be performed to assess the efficacy and safety of this laser and to better understand its mechanism of action in promoting hair regrowth.

### Fractional carbon dioxide (FRCO2) laser

The FRCO2 laser is an advanced laser system designed for various dermatological procedures and is notably recognized for its efficacy in skin resurfacing and rejuvenation. It functions primarily via fractional photothermolysis, which divides the laser beam into numerous minuscule, closely positioned microthermal zones [38]. These microthermal zones are then delivered to the skin’s surface, creating microscopic injuries known as microthermal treatment zones (MTZs) [38]. Ultimately, this laser targets these MTZs with high-intensity energy, inducing controlled thermal damage to the skin’s layers. This process stimulates the skin’s physiologic healing response, increasing the production of new collagen and elastin fibers [38, 39]. Although it is primarily known for its applications in dermatology, the FRCO2 laser has also piqued interest in the context of AA and AGA.

One documented study investigating the FRCO2 laser in this context aimed to evaluate the effectiveness and safety of the laser treatment, followed by applying topical triamcinolone spray in ten patients with treatment-resistant AA. All the patients received a total of four to eight treatment sessions, with each session being repeated at three to four-week intervals. Treatment response was assessed utilizing a quartile physician evaluation scale, categorizing the outcomes as follows: excellent (indicating regrowth exceeding 75%), good (reflecting regrowth between 50% and 75%), fair (indicating a response ranging from 26 to 50%), or poor (denoting regrowth less than 25%). Seven of the eight patients who successfully underwent the treatment regimen fully recovered the treated area. On the other hand, one patient exhibited limited improvement, even after completing four sessions. Importantly, no significant adverse effects were observed among patients [40].

Another recent study assessed the effectiveness of three approaches in the context of AA treatment: FRCO2 monotherapy, FRCO2 combined with topical betamethasone valerate cream, and topical betamethasone valerate cream monotherapy. The study involved 30 adult patients, both male, and female, with AA and excluded individuals under 18, pregnant or lactating women, those with alopecia totalis (AT) or alopecia universalis, those who had received recent topical or systemic treatment, and those with a history of hypertrophic scars or keloids. Patients were divided into three groups: Group A received FRCO2 laser treatment, group B received FRCO2 Laser along with betamethasone valerate cream, and group C received betamethasone valerate cream alone for four months. In Group A, there was a significant reduction in the Severity of Alopecia Tool (SALT) score, a decrease in dystrophic hair, and an increase in terminal hair. Hair regrowth began for eight patients after the third and two after the fifth sessions. Group B showed a significant decrease in SALT score, a reduction in dystrophic hair, and an increase in terminal hair. One patient saw a visible response after the first session, eight after the third session, and one after the fourth session. Finally, group C demonstrated a significant decrease in SALT score, dystrophic hair reduction, and an increase in terminal hair. Six patients showed a visible response after four weeks, three after six weeks, and one after eight weeks. These results highlight that the FRCO2 laser, whether used as monotherapy or combination therapy, is a safe and effective treatment for localized AA. Several studies in the current literature support this, but conducting additional studies in the future would be ideal for further advancing our understanding and knowledge of the long-term effects of this treatment [41].

In the context of FRCO2 for AGA treatment, one study examined the effectiveness of hair regrowth factors alone or in combination with ablative FRCO2 laser for male AGA. A randomized half-split approach was used on 28 participants, treating one side of the scalp with the FRCO2 laser and both sides with hair regrowth factors over six sessions with two-week intervals. Evaluations included global photographs, dermoscopy assessments, and scanning electron microscopy to examine hair follicle phase and hair-shaft diameter changes at baseline and four months post-treatment. Both the combined treatment group and the growth factor group alone showed significant increases in hair density: mean hair density increased from 114 ± 27 to 143 ± 25/cm2 (*P* < 0.001) in the combined group and from 113 ± 24 to 134 ± 19/cm2 in the growth factor group (*P* < 0.001). However, the combined group exhibited a more remarkable mean change from baseline. Global photographs displayed improvement in 93% (25/27) patients in the combined group and 67% (18/27) patients in the growth factor group. Scanning electron microscopy revealed transitions from telogen to anagen phase in hair follicles and increased hair-shaft diameter in some patients. These findings suggest that the combination of ablative fractional CO2 laser and hair regrowth factors may be a viable alternative for treating male AGA, especially in individuals who are not suitable candidates or would prefer to avoid surgical intervention [42].

### Narrowband ultraviolet B phototherapy (NB-UVB)

NB-UVB has established its prominence as a therapeutic modality in various dermatologic conditions. This treatment employs a narrow wavelength range, typically between 311 and 313 nm, either in generalized or targeted applications, and has found utility in diverse conditions such as vitiligo, psoriasis, eczema, mycosis fungoides, pruritus, and more [43]. Mechanistically, NB-UVB penetrates the epidermis and superficial dermis, playing a role in immune suppression, cell cycle arrest, and modulation of cytokine expression [44]. Given its immune-modulating properties, there is a growing interest in exploring the potential of NB-UVB in managing AA.

Between 1996 and 2002, Jury et al. evaluated the use of NB-UVB in multiple dermatoses on patients under 16 years old through a retrospective review. Six patients were treated for AA with targeted NB-UVB to the scalp for a median course of 20 treatments with incremental dosage increases of 20% per treatment. Results showed no improvement in five out of six patients. Adverse effects were limited to erythema in 30% of patients. This study is limited by its low sample size of AA patients and lack of a standardized phototherapy administration protocol [45].

A subsequent retrospective review (2004–2009) encompassed 25 patients diagnosed with AA who underwent NB-UVB phototherapy. Patients were categorized based on the extent of hair loss, with “extensive patchy hair loss (*n* = 15)” and “entire scalp hair loss (*n* = 10)” groups analyzed separately. The mean number of phototherapy sessions and cumulative dosage were 46.4 and 63.9 J/cm^2, respectively. In the extensive patchy hair loss group, only 22.2% of patients exhibited an excellent response (> 75% hair regrowth), while 53.3% experienced a poor response (< 25% hair regrowth). The entire scalp hair loss group demonstrated a similar trend, with 20% achieving an excellent response and 40% experiencing a poor response. These results indicate varying degrees of efficacy. Concomitant systemic corticosteroid uses in eight patients appeared to influence treatment outcomes, with a significant difference observed in patients achieving an excellent response. Consequently, the authors concluded that despite its generally favorable tolerability, NB-UVB does not emerge as an efficacious treatment for AA [46].

A more recent retrospective review (2014–2016) conducted by Salman et al. encompassed 173 patients with various dermatoses, including 32 cases of AA, treated with targeted NB-UVB. The average number of treatment sessions and cumulative dosage were 23.8 and 18.2 J/cm^2, respectively. Among AA patients, 88.2% had previously used topical treatments without satisfactory results. The treatment outcomes were assessed based on hair regrowth, revealing that 40.6% of patients achieved moderate or better improvement (> 50% hair regrowth), with 28.1% experiencing a complete response (> 90% hair regrowth). When cases lost to follow-up were excluded, 52.9% of patients responded utterly. These results indicate that a prolonged treatment course may challenge achieving satisfactory results. The authors attribute their higher efficacy to using targeted phototherapy over a generalized approach. Mild erythema was seen in 52.9% of cases, whereas severe adverse reactions were rare [47].

These studies collectively suggest that NB-UVB is generally a safe intervention that confers some benefit in the context of AA. However, definitive conclusions still need to be made available due to several limitations inherent in these retrospective investigations. El Taieb et al. conducted a RCT between 2017 and 2018 to assess topical calcipotriol’s efficacy and NB-UVB in treating scalp AA. The study enrolled 60 patients with AA, and they were divided into four groups of 15: topical calcipotriol, NB-UVB phototherapy, topical calcipotriol and NB-UVB, and placebo. The primary outcome measured was the SALT score, which quantifies the extent of hair loss—a standardized protocol comprised of two weekly phototherapy sessions over three months. The study’s results indicated that NB-UVB phototherapy was effective for scalp AA during the 3-month treatment period. Patients in the NB-UVB alone group showed significant improvement in their after-treatment SALT score compared to the placebo group. This finding demonstrates the potential of NB-UVB phototherapy as a valuable therapeutic option for patients with scalp AA. The authors did not find a significant improvement in NB-UVB plus topical calcipotriol over NB-UVB alone. The study hypothesizes that the benefit of NB-UVB is derived from its immunomodulatory effects and its positive effects on serum vitamin D3 [48].

As such, NB-UVB phototherapy shows promise as a therapeutic treatment for AA. Yet, the literature remains riddled with limitations, including the absence of randomization, the lack of control groups, and the potential confounding factor of spontaneous remission, which may complicate the discernment of the therapeutic cause leading to satisfactory results. Moving forward, further RCTs are warranted to provide more robust insights into the utility of NB-UVB in AA management. Additionally, future research endeavors should consider evaluating combination therapies that incorporate medical interventions to optimize treatment outcomes.

### Ultraviolet A-1 phototherapy (UVA1)

UVA1 phototherapy has demonstrated significant advantages in treating dermatologic conditions affecting both the epidermis and dermis [49]. As a treatment that offers minimal systemic side effects, UVA1 phototherapy has proven to penetrate the deeper layers of the skin to cause apoptosis in T-cells and proliferate endothelial cells to increase neovascularization [50]. These distinctive properties position UVA1 phototherapy as a promising treatment for several dermatologic skin conditions, including AA.

A study by Herz-Ruelas et al. aimed to explore the therapeutic potential of UVA-1 phototherapy in patients with unresponsive AA by determining the appropriate dosimetry and assessing its effects. 13 men and nine women were recruited for this study. Each participant was administered 25 sessions of 30 J/cm^2. If hair regrowth was < 75%, the dose was increased to 60 J/cm^2. If hair improvement remained < 75%, 25 sessions at 120 J/cm^2 were administered. If complete hair regrowth occurred before 75 sessions, patients underwent a final visit for biopsies and evaluation using the Severity of Alopecia Tool (SALT). Results revealed that nine patients achieved complete hair regrowth (S0), eight achieved significant improvement (S1), and five showed marked progress (S4), with the most pronounced improvement observed in those who received the 60 J/cm^2 treatment. Anagen hairs increased by 43.75%, while telogen and catagen hairs decreased by 16.3% and 22.7% respectively. The benefits of UVA-1 phototherapy persisted for six months post-treatment, with minimal side effects, including mild xerosis and hyperpigmentation in six cases [51].

While this study’s results offer valuable insights into the potential benefits of UVA1 phototherapy for patients with unresponsive AA, further research studies are necessary to comprehensively evaluate its effectiveness, safety, and long-term outcomes within this patient population.

### Neodymium: Yttrium-Aluminum-Garnet (nd: YAG 1,064 nm)

The Nd: YAG 1064 nm laser, also known as the Neodymium-doped Yttrium Aluminum Garnet laser emitting light at a wavelength of 1064 nanometers, is commonly known for its versatility and effectiveness in treating a wide range of skin conditions and performing various cosmetic and therapeutic procedures [52, 53]. It operates at a wavelength that can penetrate the skin deep enough to reach the hair follicles while minimizing damage to the surrounding tissue; because of this characteristic, this laser’s thermal energy can stimulate dormant hair follicles and potentially promote hair regrowth in affected areas. While research in this realm is ongoing, early studies and clinical observations suggest that the Nd: YAG 1,064 nm laser may have a role in the management of AA, offering hope to individuals dealing with this distressing condition.

One case study reported a 25-year-old female who presented with AT, the most severe presentation of AA, affecting her scalp for the past two years. Traditional topical and ILCS treatments were initiated on both sides of the scalp, serving as a control on the left half. The right half of the scalp involved the application of the 1064 nm Nd: YAG picosecond laser at monthly intervals. Following six treatment sessions, the right side, treated with the 1064 nm picosecond Nd: YAG laser, exhibited significantly more significant improvement compared to the left side, treated solely with steroids [54].

The results demonstrated in the study discussed above suggest that the 1064 nm ps-Nd: YAG laser holds potential as a non-invasive and promising therapeutic option for AA. However, there currently needs to be more documented studies that have assessed the effects of this laser for the treatment of AA; thus, to establish its effectiveness, safety, and long-term outcomes more conclusively and generalize its application, further investigations and clinical trials are necessary.

## Discussion

Alopecia is a highly prevalent condition that can be highly distressing to patients, necessitating continued improvement in the current treatment modalities to increase the efficacy of management while minimizing side effects. Most studies discussed in this review have shown their efficacy in improving hair regrowth and density in alopecia patients. However, caution must be considered when interpreting the findings of these results, as some laser devices lack robust clinical data. In addition, it is important to highlight that irradiation of the skin with lasers may generate reactive oxygen species which is likely the cause of many side-effects experienced after laser treatments, such as erythema and pigmentation [55–57]. Further studies are needed to assess the efficacy, safety profile, and mechanism of action better. Nonetheless, the current findings in the literature indicate that laser-based therapies hold great potential for hair regrowth, and clinicians should consider incorporating them when managing patients suffering from alopecia.
